# Benefits of Splenectomy and Curative Treatments for Patients with Hepatocellular Carcinoma and Portal Hypertension: a Retrospective Study

**DOI:** 10.1007/s11605-018-3981-9

**Published:** 2018-12-03

**Authors:** Youliang Pei, Songshan Chai, Yuxin Zhang, Zhanguo Zhang, Xiaoping Chen, Wanguang Zhang

**Affiliations:** Hepatic Surgery Center, Tongji Hospital, Tongji Medical College, Huazhong University of Science and Technology, Wuhan, China

**Keywords:** Hepatocellular carcinoma, Portal hypertension, Curative treatment, Splenectomy

## Abstract

**Background:**

We aimed to explore the benefit of splenectomy combined with curative treatments (liver resection or local ablation) for patients with hepatocellular carcinoma and portal hypertension.

**Methods:**

The records of 239 patients with hepatocellular carcinoma and portal hypertension undergoing either splenectomy combined with liver resection or local ablation were reviewed retrospectively. Perioperative complications and survival outcome were evaluated, and liver function 1 year later was reassessed according to the Child score.

**Results:**

The post-hepatectomy liver failure rates and 30-day mortality were 3.3% and 2.1%, respectively. The 1-, 3-, and 5-year overall survival rates were 95.1%, 73%, and 47.5% for patients with Child grade A and 92.2%, 51.2%, and 19.8% for Child grade B, respectively. The median survival time for patients with Child scores of 5, 6, 7, 8, and 9 were 61.5, 51.3, 44.8, 33.7, and 23.4 months, respectively. After multivariable analysis, tumor size, tumor number, post-hepatectomy liver failure, and Child score were independent risk factors for overall survival. Liver function was converted to Child grade A in 98 of 101 patients (97%) who had preoperative Child grade B 1 year after splenectomy.

**Conclusion:**

Patients with hepatocellular carcinoma and portal hypertension can benefit from splenectomy combined with curative treatments, especially those with Child scores of 5, 6, and 7. Liver function improved significantly 1 year after splenectomy in patients with preoperative Child grade B.

**Electronic supplementary material:**

The online version of this article (10.1007/s11605-018-3981-9) contains supplementary material, which is available to authorized users.

## Introduction

Cirrhosis, portal hypertension (PH), and hepatocellular carcinoma (HCC) are inseparable and mutually influence each other.^1^ Patients with HCC and PH usually have impaired liver function, excessive portal blood flow and pressure, high risk of bleeding tendency, and poor performance status, which both affect the prognosis and limit the opportunity for most curative treatment modalities for HCC, precluding subsequent treatment options in recurrent patients, including liver resection, local ablation, and transarterial chemoembolization (TACE).^2,3^ Theoretically, liver transplantation seems to be the optimal method to simultaneously cure the tumor and replace the cirrhotic liver for patients with early stage HCC and severe cirrhosis.^4^ However, due to the shortage of liver donors, only a small proportion of patients can successfully undergo liver transplantation. Alternative treatment deserves to be explored for this specific group of patients.

According to the European Association for the Study of Liver (EASL) and the American Association for the Study of Liver Diseases (AASLD) treatment guidelines for HCC, PH and decompensated liver function are considered contraindications for liver resection due to a very high risk of post-hepatectomy liver failure (PHLF) and mortality.^2,3^ However, remarkable advances in preoperative evaluation, surgical techniques, and perioperative care have reinforced the role of liver resection for patients with HCC and PH.^5–7^ Even in some selective candidates with significant PH, liver resection can receive a long-term survival and relatively low perioperative mortality. Local ablation has been reported as an alternative to liver resection for patients with small HCC (tumor diameter less than 3 cm) or those who cannot endure a resection and have achieved long-term survival.^8–10^ After curative treatment for HCC, two other lethal factors in patients with HCC and PH are variceal bleeding resulting from the development of PH and the decompensation of liver function.^11^ Splenectomy is an effectively preventable measurement to reduce the portal flow and decrease the tendency of variceal bleeding, and it can also improve the liver function in some decompensated patients.^12–14^

Several studies have also reported that synchronous splenectomy and liver resection or splenectomy combined with local ablation in patients with HCC and splenomegaly achieve both short-term and long-term survival.^15–21^ However, patients enrolled in these studies were heterogeneous, either with good liver function reserve or with large tumor burden. In this study, we enrolled more cirrhotic patients with limited tumor burden and PH who were treated by synchronous splenectomy and curative treatment, and both the short- and long-term outcomes, including perioperative mortality and morbidity, the liver function test 1 year after surgery, and long-term follow-up, were evaluated to identify who could benefit from this surgical procedure.

## Material and Methods

### Patients and Data Collection

We retrospectively reviewed the data for all patients who received surgical treatment for HCC and splenectomy for PH at the Department of Hepatic surgery center of Tongji Hospital during January 2005 and December 2015. The clinical data for all patients were prospectively collected from electronic medical records. This study was conducted by the Declaration of Helsinki and approved by the Clinical Research Ethics Committee of the Tongji Hospital.

### Preoperative Assessment and Surgical Indication

Preoperative evaluation included the liver- and tumor-related test, general condition assessment. All patients underwent detailed laboratory evaluation, including the liver and renal function test, coagulative function test, the complete blood count, serum levels of α-fetoprotein (AFP), and indocyanine green retention rates at 15 min (ICG-R15). The Child–Turcotte–Pugh (hereinafter called Child) score and the model for end-stage liver disease (MELD) score were calculated as previously described.^22^ Abdominal ultrasonography was performed for all patients to obtain necessary information about the tumor. Once a suspicious nodule was detected, additional contrast-enhanced computed tomography (CT) and/or dynamic contrast-enhanced magnetic resonance imaging (MRI) was performed to confirm the diagnosis of HCC. Upper gastrointestinal endoscopy was performed for all patients to evaluate the presence of varices, which was the primary evidence of PH. Even though the measurement of the hepatic venous pressure gradient is the gold standard to detect the presence of PH, it was not performed routinely in our center. Other clinical data, such as splenomegaly and platelet count, were also indirect evidence to assess the presence of PH and the severity of varices. PH was indirectly defined as (1) the presence of esophageal varices detected by preoperative endoscopy screening or (2) the coexistence of splenomegaly (thickness more than 4.0 cm on transcutaneous ultrasonograph) with a platelet count < 100,000/mm^[3.2,17^

The surgical indication for HCC followed traditional guidelines but was also beyond the recommendations when the patient could not receive liver transplantation, or the surgical removal of the tumor could achieve relatively long-term survival compared with other available options, such as TACE or sorafenib. The surgical indication was limited tumor burden and a liver function of Child grade A or B, and good general condition (ECOG score < 2) and vital organ function with the ability to tolerate general anesthesia. Some patients underwent microwave ablation instead of liver resection in the following situations: (1) multiple tumors with a tumor number ≤ 3 and diameter of the largest tumor ≤ 3 cm; (2) a solitary tumor in the depth of liver parenchyma and severe cirrhosis with a Child grade B liver function and an inability to tolerate the strike of the liver resection or (4) coagulation disorders.^10^ Patients with solitary tumor adjacent to major vascular or biliary structure were also the candidates for liver resection rather than local ablation to avoid the impairment of thermal injury during ablation. In addition, some patients underwent additional Hassab’s surgery (splenectomy combined with pericardial devascularization) at the same time due to a history of variceal bleeding or the presence of large varices detected by endoscopy or CT scan.^21^ Patients with a liver function of Child grade B received medical treatment until the liver function improved to Child grade A before the operation. No patient in the present study was treated with emergency surgery.

### Intraoperative Management

Intraoperative management was described as follows. All operations were carried out under general anesthesia and performed by experienced surgeons in open and laparoscopic splenectomy and hepatectomy. Usually, the liver resection or local ablation was done before the splenectomy. Liver parenchyma transection and splenectomy were conducted with the use of an ultrasonic scalpel. The Pringle maneuver was not routinely used; it was used only when uncontrolled bleeding occurred. Intraoperative ultrasound was performed to detect additional tumor nodules not revealed preoperatively or to direct the local ablation. Major hepatectomy was defined as removal of three or more Couinaud liver segments, and minor hepatectomy as removal of fewer than three segments.

### Postoperative Care and Assessment

After surgery, intravenous broad-spectrum antibiotics were applied to prevent infectious complications. If an infectious complication was detected, antibiotics that were sensitive to the cultured pathogen were applied. If there was no active intraperitoneal bleeding or the color of abdominal drainage fluid was normal, prophylactic anticoagulation therapy was started by subcutaneous injection of low-molecular-weight heparin (LMWH) to prevent the development of postoperative portal vein thrombosis (PVT) on the third day, regardless of whether PVT was detected. Ultrasonography or CT scan was routinely done on the third day after surgery to detect the presence of PVT, ascites, and hydrothorax. Postoperative complications were categorized using the Clavien-Dindo classification of surgical complications, and major complication was defined as Clavien-Dindo grade ≥ 3.^23^ PHLF was defined according to the “50–50 criteria” on the fifth day after the operation.^24^ If PVT, ascites, or hydrothorax was detected, ultrasonography or CT scan was repeated to evaluate the therapeutic response. These complications were assessed daily from the day of surgery until discharge.

### Follow-up

All patients were regularly followed with serum AFP level, liver function test, and ultrasonography or CT scan every 1–3 months during the first 2 years after surgery. The intervals were extended to 3–6 months after that. Investigation with control-enhanced CT or MRI scan was performed if ultrasonography suspected tumor recurrence. Once intrahepatic recurrence was identified, the liver- and tumor-related data were reassessed according to the EASL and AASLD treatment guidelines for HCC. Repeated liver resection, local ablation, TACE, or ethanol injection was performed based on the reassessment results. Persistent antiviral therapy with entecavir was recommended for patients with preoperatively active hepatitis. In addition, a prophylactic anticoagulation therapy with LMWH to prevent the development of PVT was extended to 3 months after discharge. The median follow-up time was 43.1 months, with a range from 32 to 61 months.

### Statistical Analysis

Continuous variables are expressed as the median and interquartile range (IQR). Categorical variables are reported as the number of cases and prevalence. Liver function change was compared using the paired-samples *t* test procedure. Patients’ survival curves were computed using the Kaplan–Meier method and compared using the log-rank test. Disease-free survival (DFS) was measured from the date of operation until the detection of tumor recurrence. Overall survival (OS) was defined as the interval between the date of operation and the date of tumor-related death; patients who died from other causes were defined as a censor. Factors influencing OS were analyzed using multivariate analysis with Cox’s proportional hazard model. We used two-sided *P* values of < 0.05. *P* < 0.05 was considered statistically significant. All statistical analyses were performed with SPSS® version 19.0 (IBM, Armonk, New York, USA) or Prism 5 (GraphPad Software, Inc., La Jolla, CA).

## Results

**Fig. 1:**
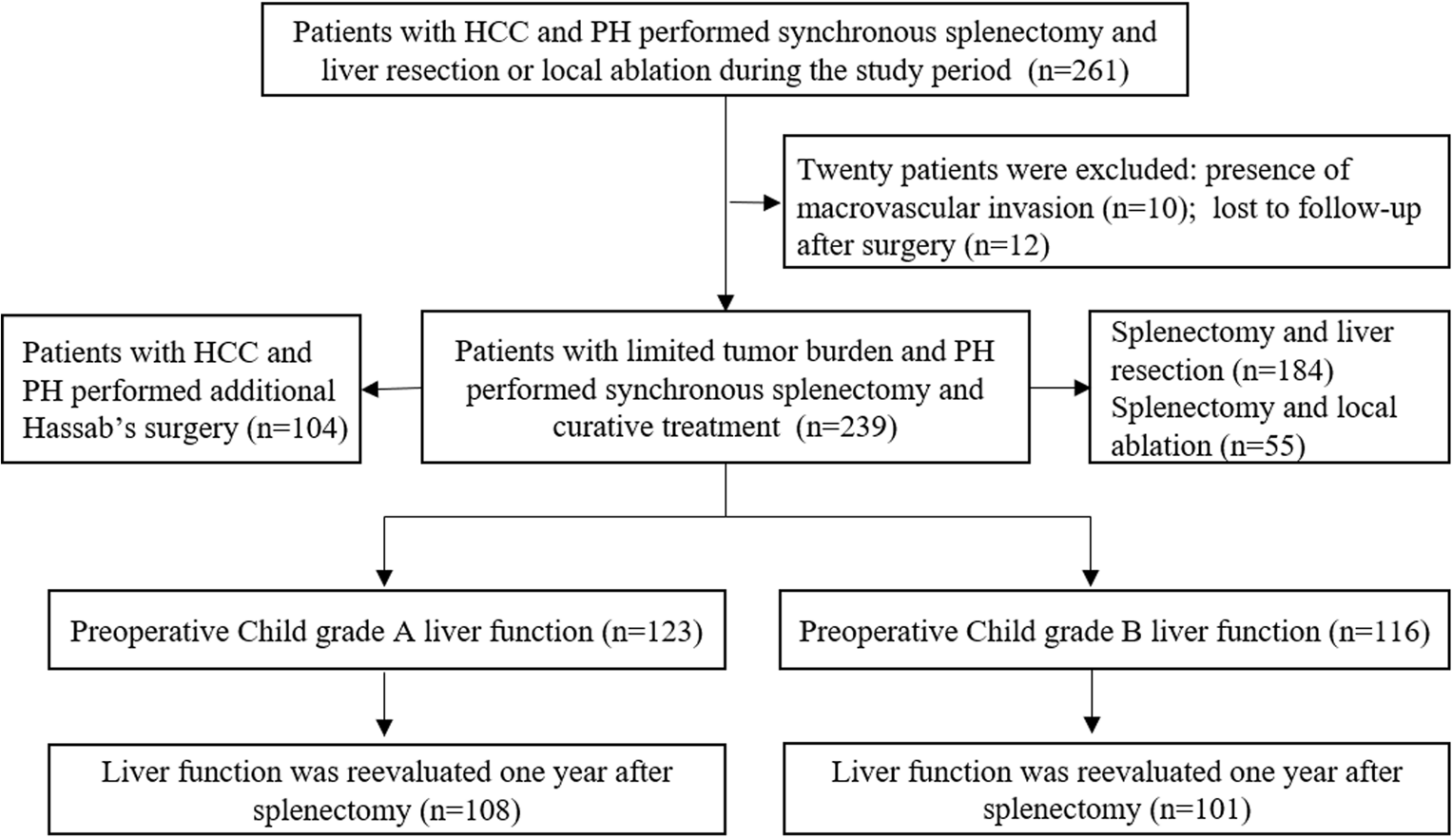
Study profile

During the study period, clinical data of 261 patients with HCC and PH who received surgical treatment for HCC and splenectomy for PH were retrieved; 22 patients were excluded from this study: 10 patients were presented with macrovascular invasion, and 12 patients lost follow-up. Finally, 239 consecutive patients with HCC and PH who underwent simultaneous splenectomy and curative treatment were included in this study (Fig. 1).

### Baseline Characteristics

The basic preoperative information for all patients is listed in Table 1. Among these patients, 189 (79.1%) were male, 50 (20.1%) were female, and the median age was 50 (IQR 42–58) years. A total of 52 patients (21.8%) had a history of variceal bleeding, 40 (16.7%) needed additional transfusion due to a large volume of blood loss, and only 9 (3.8%) received preoperative endoscopic therapy (sclerotherapy or band ligation). The number of patients with small varices was 100 (41.9%), median 46 (19.2%), and large 93 (38.9%). Most patients (221 [92.5%]) presented with severe cirrhosis; only a small portion of patients (18 [7.5%]) had mild cirrhosis. Median MELD and Child scores were 6 (IQR 5–7) and 10.1 (IQR 7.6–12.2), respectively. Median serum AFP was 43.6 (IQR 7.9–496) ng/ml, and the median ICG-R15 was 15.6% (IQR 5.6–29.4%). The number of patients with decompensated liver function indicated by Child B classification was 116 (48.2%), and well-preserved liver function with Child A classification was 123 (51.8%). Median spleen thickness and portal vein diameter were 5.3 cm (IQR 4.7–5.8) and 1.3 cm (IQR 1.2–1.5), respectively. The median size of the largest tumor nodule was 3 cm (IQR 2.5–4.4); 222 patients (92.9%) had a single tumor, and only17 patients (7.1%) had multiple tumors.

**Table 1:** Basic preoperative characteristics of 239 patients underwent curative treatment for HCC and splenectomy for PH

Characteristic	No. (%) of patients
Age, median (IQR), year	50 (42–58)
Sex	
Male	189 (79.1)
Female	50 (20.9)
Preoperative variceal bleeding	52 (21.8)
Preoperative transfusion	40 (16.7)
Preoperative endoscopy therapy	9 (3.8)
Varices	
Small	100 (41.9)
Median	46 (19.2)
Large	93 (38.9)
Cirrhosis	
Mild	18 (7.5)
Severe	221 (92.5)
Presence of ascites	110 (46.0)
White blood cell count (IQR), *10^12^/L	2.7 (2.1–3.6)
Platelet count (IQR), *10^3^/mm^3^	50 (36–67)
Albumin (IQR), g/L	35.8 (32.9–39.5)
Total bilirubin (IQR), μmol/L	17.3 (13.1–24.3)
PT (IQR), second	15.5 (15.3–15.8)
AFP (IQR), ng/mL	43.6 (7.9–496)
ICG R-15 (IQR), %	15.6 (5.6–29.4)
MELD score (IQR), point	10.1(7.6–12.2)
Child score (IQR), point	6 (5–7)
Child classification	
A	123 (51.5)
B	116 (48.2)
Child score 5/6/7/8/9	89/34/82/29/5
ECOG score	
0	206 (81.2)
1	33 (13.8)
Median splenic thickness (IQR), cm	5.3 (4.7–5.8)
Median portal vein diameter (IQR), cm	1.3 (1.2–1.5)
Largest tumor diameter (IQR), cm	3.0 (2.5–4.4)
> 3	125 (52.3)
≤ 3	114 (47.7)
Tumor number	
Solitary	222 (92.9)
Multiple	17 (7.1)

*HCC*, hepatocellular carcinoma; *PH*, portal hypertention; *IQR*, interquartile ranges; *PT*, prothrombin time; *AFP*, α-fetoprotein; *ICG R-15*, indocyanine green retention rates at 15 min; *MELD*, Model for End-State Liver Disease; *ECOG*, Eastern Cooperative Oncology Group

### Perioperative Data and Postoperative Complications

Table 2 summarizes the main variables of intraoperative and postoperative outcomes. Laparoscopic surgery was performed for 32 patients (13.4%) and open surgery for 207 patients (86.6%). Most patients (184 [77%]) underwent splenectomy and hepatectomy at the same time, whereas a relatively small portion of patients (55 [23%]) underwent splenectomy and local ablation therapy; 104 patients (43.5%) received additional Hassab’s operation; 31 resections (16.8%) were anatomical, and only 5 (2.7%) were major resections. Median operative time was 246 min (IQR 213–295); intraoperative estimated blood loss was 300 ml (IQR 200–600); 112 patients (46.9%) received an intraoperative transfusion, and median transfusion volume was 800 ml (IQR 550–1000). The Pringle maneuver was applied in 107 patients (44.8%), and the median blocking time was 14 min (IQR 10–16). The median length of hospital stay was 15 days (IQR 13–20).

**Table 2:** Perioperative data and postoperative complications

Variable	No. (%) of patients
Operative approach	
Laparoscopic surgery	32 (13.4)
Open surgery	207 (86.6)
Hepatectomy + splenectomy	184 (77.0)
Local ablation + splenectomy	55 (23.0)
Hassab’s surgery	104 (43.5)
Anatomical hepatectomy^†^	31 (16.8)
Major hepatectomy^†^	5 (2.7)
Operative time (IQR), min	246 (213–295)
Intraoperative blood loss, ml	300 (200–600)
Intraoperative blood transfusion	112 (46.9)
Transfusion volume, ml	800 (550–1000)
Pringle maneuver	107 (44.8)
Duration, min	14 (10–16)
In-hospital stay (IQR), days	15 (13–20)
Complication classification	180 (75.3)
Minor	122 (51.0)
Major	58 (24.3)
Complication type	
General	
Thoracentesis	38 (15.9)
Renal dysfunction	4 (1.7)
Variceal rapture	5 (2.1)
Surgical	
Postoperative transfusion	140 (58.6)
PVT	65 (27.2)
Pancreatic fistula	16 (6.7)
Intra-abdominal hemorrhage	17 (7.1)
Wounding infection	9 (3.8)
Reoperation	6 (2.5)
Liver-related	
Bile leakage	3 (1.3)
Transient PHLF	8 (3.3)
Early mortality	5 (2.1)

*IQR*, interquartile ranges; *PVT*, portal vein thrombosis; *PHLF*, post-hepatectomy liver failure

^†^Anatomical and major hepatectomy counted only in patients who underwent liver resection (*n* = 184)

The postoperative complications are shown in Table 2. The overall complication rate is 75.3% (180/239), and 58 patients (24.3%) developed major complication. The most common complication is postoperative transfusions, 140 patients (58.6%) required postoperative transfusions due to hypoproteinemia and poor coagulation. In addition, 38 patients (15.9%) had to undergo thoracentesis under local anesthesia due to refractory pleural effusion. Even though the total morbidity is very high; the lethal complications are relatively low, eight patients (3.3%) developed PHLF; six patients (2.5%) underwent reoperation for uncontrolled intra-abdominal hemorrhage. Five patients (2.1%) died within 30 days, three from liver failure, one from abdominal infection, and one from intra-abdominal hemorrhage. Variceal bleeding from varices rupture during hospital stay was observed in 5 patients (2.1%). PVT was detected in 65 patients (27.2%).

### Survival Benefit from Splenectomy and Curative Treatment

**Fig. 2:**
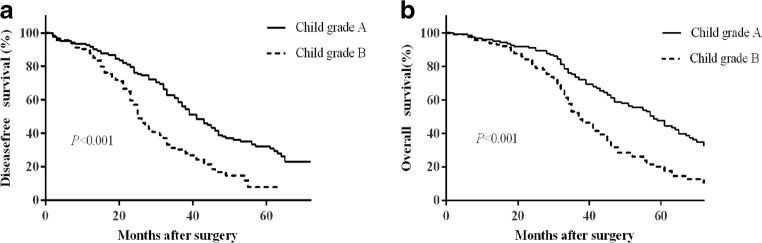
Survival curve for patients with different Child grade. **a** Disease-free survival and **b** overall survival (both *P* < 0.001)

**Fig. 3:**
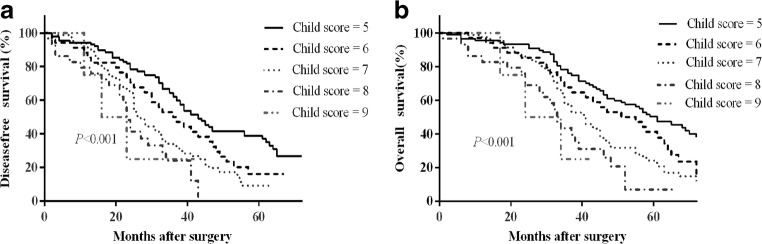
Survival curve for patients with different Child scores. **a** disease-free survival and **b** overall survival (both *P* < 0.001)

To evaluate the effect of liver function on the prognosis, we compared the baseline characteristics of patients with different Child grade (Supplemental Table). Patients with Child grade B had significant poor liver function reserve, such as lower serum albumin, higher ICG R-15, more severe PH, whereas the tumor-related data had no statistic difference in the two subgroups. The largest tumor size and the tumor number were comparable in the two subgroups. The DFS rates at 1, 3, and 5 years after surgery were 91.1%, 58.2%, and 37.2%, respectively, for patients with a liver function of Child grade A and were 87.9%, 29.8%, and 7.6% for patients with a liver function of Child grade B (*P* < 0.001; Fig. 2a). The OS rates at 1, 3, and 5 years after surgery were 95.1%, 73.0%, and 47.3%, respectively, for patients with a liver function of Child grade A and were 92.2%, 51.2%, and 19.8% for patients with a liver function of Child grade B (*P* < 0.001; Fig. 2b). Among patients with a liver function of Child grade A, the median survival time after surgery was 20 months longer than that for patients with a liver function of Child grade B (57 months [95% CI, 49.3–64.7] vs. 37 months [95% CI, 32.4–41.6]; *P* < 0.001) (Fig. 2b). Patients with a Child A classification achieved significantly better DFS rates, OS rates, and median survival time than those with a Child B classification. The median survival time after surgery according to the Child scoring system was as follows: 5 points, 59 months (95% CI 49.2–68.8); 6 points, 51 months (95% CI 33.9–68.1); 7 points, 41 months (95% CI 35.2–46.8); 8 points, 33 months (95% CI 29.3–36.7); 9 points, 24 months (95% CI 8.9–39.1), *P* < 0.001, (Fig. 3a, b). Patient with a Child score of 5, 6, and 7 had significantly longer DFS and OS.

### Prognostic Factors for Patients with HCC and PH

Prognosis factors were analyzed for variables using a Cox proportional hazards model. In univariable analysis, ECOG score beyond 0 (hazard ratio (HR) 1.623, 95% CI 1.052–2.506; *P* = 0.029), tumor size larger than 3 cm (HR 2.077, 95% CI 1.052–2.506; *P* < 0.001), multiple tumors (HR 2.016, 95% CI 1.182–3.437; *P* = 0.010), ICG R15 > 20% (HR 1.581, 95% CI 1.173–2.132; *P* = 0.003), PHLF (HR 4.248, 95% CI 2.068–8.724; *P* < 0.001), Child score 7 vs. 5 (HR 1.953, 95% CI 1.369–2.787, *P <* 0.001), Child score 8 vs. 5 (HR 3.265, 95% CI 1.996–5.340, *P* < 0.001), and Child score 9 vs. 5 (HR 4.391, 95% CI 1.360–14.185, *P* = 0.013) were prognostic factors for worse overall survival (Table 3). For multivariable analysis, Cox regression analysis was performed in a backward manner. Tumor size larger than 3 cm (HR 1.821, 95% CI 1.305–2.543; *P* < 0.001), multiple tumors (HR 2.182, 95% CI 1.249–3.809; *P* = 0.006), PHLF (HR 4.538, 95% CI 2.106–9.781; *P* < 0.001), Child score 7 vs. 5 (HR 2.322, 95% CI 1.408–3.830, *P =* 0.001), Child score 8 vs. 5 (HR 4.803, 95% CI 2.489–9.269, *P <* 0.001), and Child score 9 vs. 5 (HR 8.618, 95% CI 2.390–31.068, *P* = 0.001) remained independent risk factors for worse overall survival (Table 3).

**Table 3:** Univariate and multivariate analyses of prognostic factors for overall survival

Variable	Univariate analyses		Multivariate analyses	
	HR (95%CI)	*P* value	HR (95%CI)	*P* value
Preoperative variceal bleeding (yes vs. no)	1.130 (0.793–1.161)	0.447		
ASA score (> 2 vs. ≤ 2)	1.242 (0.720–2.146)	0.434		
ECOG score (1 vs. 0)	1.623 (1.052–2.506)	0.029	1.161 (0.710–1.900)	0.552
Varices				
Median vs. small	0.630 (0.454–0.874)	0.106		
Large vs. small	0.780 (0.527–1.154)	0.214		
Cirrhosis (severe vs. mild)	1.677 (0.885–3.178)	0.113		
Child-Pugh score, point				
6 vs. 5	1.407 (0.898–2.203)	0.136	1.507 (0.956–2.376)	0.078
7 vs. 5	1.953 (1.369–2.787)	< 0.001	2.322 (1.408–3.830)	0.001
8 vs. 5	3.265 (1.996–5.340)	< 0.001	4.803 (2.489–9.269)	< 0.001
9 vs. 5	4.391 (1.360–14.185)	0.013	8.618 (2.390–31.068)	0.001
Tumor size (> 3 cm vs. ≤ 3 cm)	2.077 (1.583–2.725)	< 0.001	1.821 (1.305–2.543)	< 0.001
Tumor number (multiple vs. single)	2.016 (1.182–3.437)	0.010	2.182 (1.249–3.809)	0.006
ICG R15 (> 20% vs. ≤ 20%)	1.581 (1.173–2.132)	0.003	1.161 (0.855–1.577)	0.340
Open vs. laparoscopic	1.286 (0.851–1.941)	0.232		
Surgical procedure (ablation vs. resection)	1.520 (1.084–2.130)	0.125		
Hassab’s operation (yes vs. no)	1.195 (0.893–1.600)	0.230		
PHLF (yes vs. no)	4.248 (2.068–8.727)	< 0.001	4.538 (2.106–9.781)	< 0.001
Reoperation (yes vs. no)	1.173 (0.519–2.653)	0.701		
PVT (presence vs. absence)	1.116 (0.806–1.545)	0.509		
Postoperative transfusion (yes vs. no)	1.170 (0.871–1571)	0.298		

*HR*, hazard ratio; *CI*, confidence interval; *ECOG*, Eastern Cooperative Oncology Group; *ICG R-15*, indocyanine green retention rates at 15 min; *PHLF*, post-hepatectomy liver failure

### Comparison of the Liver Function Changes 1 Year After Splenectomy

**Fig. 4:**
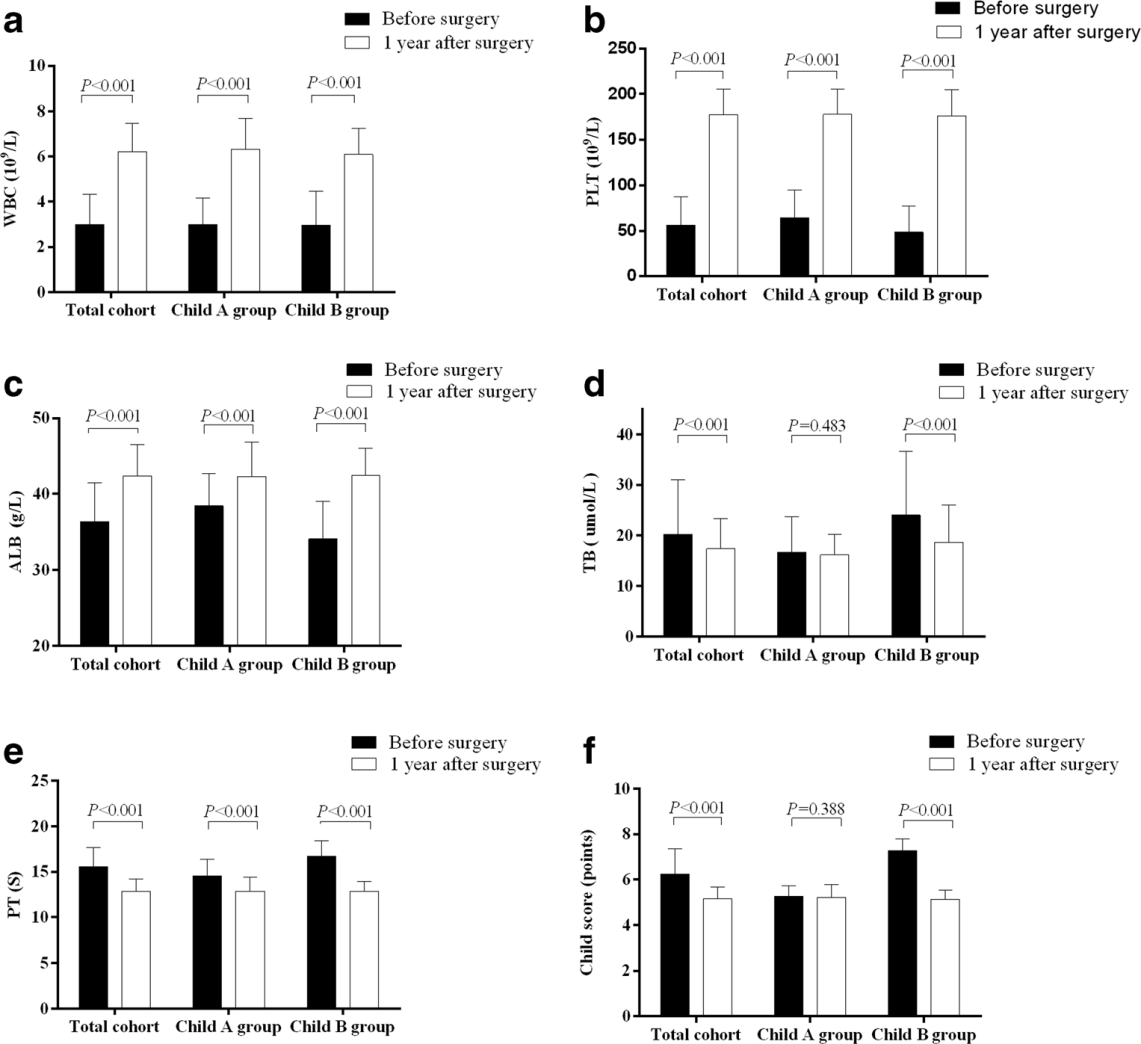
Comparison of laboratory test before and 1 year after splenectomy. Whtile blood cell (WBC) count (**a**), Platelet count (**b**), and serum albumin levels (**c**) in the whole cohort and subgroup both significantly increased in 1 year after splenectomy (*P* < 0.001), more significant in preoperative Child-B group (*P* < 0.001); Serum total bilirubin levels (**d**) decreased significantly in the whole cohort and preoperative Child-B group (*P* < 0.001), but this change did not observe in preoperative Child-A group (*P* = 0.483); Prothrombin time (**e**) in the whole cohort and each subgroup deceased significantly; Child score (**f**) decreased in the entire cohort and preoperative Child-B group (*P* < 0.001), but not significant in preoperative Child-A group (*P* = 0.338)

In addition to screening the recurrence of the tumor, we also examined patients’ liver function, coagulation function, and routine blood work 1 year after splenectomy to evaluate the influence of splenectomy on the liver function and grade it through the Child score and classification. One year after splenectomy, we successfully reassessed 209 patients’ postoperative clinical data (Table 4 and Fig. 4). Compared with preoperative liver function, liver-related laboratory parameters, such as white blood cell count, platelet count, serum albumin, total serum bilirubin, aspartate aminotransferase, alanine aminotransferase, prothrombin time, the presence of ascites, Child score, and classification, had markedly improved (*P* < 0.001). Subgroup analyses demonstrated that in patients with preoperative Child grade A (*n* = 108), total serum bilirubin decreased slightly (16.7 vs. 16.2 μmol/L, *P* = 0.483), and the mean Child score decreased somewhat (5.28 vs. 5.21, *P* = 0.388). Nine patients (8.3%) were graded as Child grade B 1 year after splenectomy. In contrast, among patients with preoperative Child grade B (*n* = 101), 98 patients (97%) had transitioned to Child grade A after splenectomy; the remaining 3 patients (3%) were still classified as Child grade B. Subgroup analysis showed that patients with a preoperative low white blood cell count, low platelet count, and low serum albumin level were substantially increased 1 year after splenectomy, while total serum bilirubin, prothrombin time, and proportion of ascites were decreased significantly. As a result, the Child score decreased significantly (7.28 vs. 5.13, *P* < 0.001) and the percentage of Child grade A increased considerably (51.7% vs. 94.7%, *P* < 0.001) 1 year after splenectomy.

**Table 4:** Liver function change before and 1 year after splenectomy

	Before splenectomy	1 year after splenectomy	*P* value
Whole cohort (*n* = 209)			
WBC count, *10^12^/L	2.99 ± 1.34	6.22 ± 1.26	< 0.001
Platelet count, *10^3^/mm^3^	56.7 ± 30.7	177.3 ± 27.85	< 0.001
Albumin, g/L	36.36 ± 5.1	42.36 ± 4.15	< 0.001
Total bilirubin, μmol/L	20.2 ± 10.8	17.4 ± 5.97	< 0.001
AST, U/L	41.1 ± 30.2	29.95 ± 16.33	< 0.001
ALT, U/L	41.1 ± 39.3	32.9 ± 18.9	0.005
Prothrombin time, second	15.6 ± 2.09	12.9 ± 1.34	< 0.001
Ascites	95(45.5)	22 (10.5)	< 0.001
Child score, points	6.24 ± 1.11	5.17 ± 0.51	< 0.001
Child grade A	108(51.7)	198(94.7)	< 0.001
Preoperative Child-A group (n = 108)			
WBC count, *10^12^/L	3.0 ± 1.15	6.33 ± 1.35	< 0.001
Platelet count, *10^3^/mm^3^	64.4 ± 30.4	177.9 ± 27.9	< 0.001
Albumin, g/L	38.5 ± 4.3	42.3 ± 4.6	< 0.001
Total bilirubin, μmol/L	16.7 ± 7.0	16.2 ± 4.1	0.483
AST, U/L	37.4 ± 25.1	29.3 ± 12.0	< 0.001
ALT, U/L	41.2 ± 44.0	31.5 ± 12.4	0.003
Prothrombin time, second	14.5 ± 1.83	12.97 ± 1.55	< 0.001
Ascites	12(11.1)	14(13.0)	0.834
Child score, points	5.28 ± 0.45	5.21 ± 0.58	0.388
Child grade A/B	108/0	100/8	0.006
Preoperative Child-B group (n = 101)			
WBC count, *10^12^/L	2.97 ± 1.51	6.1 ± 1.15	< 0.001
Platelet count, *10^3^/mm^3^	48.4 ± 28.9	176.6 ± 27.9	< 0.001
Albumin, g/L	34.1 ± 4.9	42.4 ± 3.62	< 0.001
Total bilirubin, μmol/L	24.0 ± 12.7	18.7 ± 7.3	< 0.001
AST, U/L	45.1 ± 34.4	30.7 ± 20.0	< 0.001
ALT, U/L	40.9 ± 33.6	34.5 ± 24.0	0.072
Prothrombin time, second	16.7 ± 1.73	12.85 ± 1.08	< 0.001
Ascites	83(82.2)	10(9.9)	< 0.001
Child score, points	7.28 ± 0.51	5.13 ± 0.42	< 0.001
Child grade A/B	0/101	98/3	< 0.001

*WBC*, white blood cell; *AST*, aspartate transaminase; *ALT*, alanine transaminase

### Characteristics of Tumor Recurrence and Treatments Selection After Recurrence

After surgery, the median follow-up time was 43.1 months (IQR 32–61), 197 patients experienced tumor recurrence, and 200 patients died. The most death cause is tumor recurrence (95.5%), six patients (3.0%) died from liver failure (tumor did not cause recurrent), and only one patient (0.5%) died from variceal bleeding. The most frequent recurrence pattern is intrahepatic recurrence (96.4%); 74 patients (37.6%) were diagnosed with tumor recurrence within 2 years, and 123 patients (62.4%) were beyond 2 years after the operation. After tumor recurrence, six patients performed repeat liver resection, only one patient underwent salvaged liver transplantation, 35 patients performed local ablation, 66 patients only TACE therapy, 34 patients performed ablation combined with TACE, and 54 patients received best support treatment (Table 5).

**Table 5:** Characteristics of tumor recurrence and treatment selection after recurrence

Variable	No. (%) of patients
Median follow-up period, months	43.1 (IQR 32–61)
Death	200 (83.7)
Cause of death	
Tumor recurrence	191 (95.5)
Liver failure	6 (3.0)
Variceal bleeding	1 (0.5)
Other	2 (1.0)
Recurrence	197 (82.4)
Recurrence pattern	
Intrahepatic	190 (96.4)
Extrahepatic	2 (1.0)
Both	5 (2.5)
Recurrence time	
Within 2 years	74 (37.6)
Beyond 2 years	123 (62.4)
Recurrence treatment	
Redo resection	6 (3.0)
Transplantation	1 (0.5)
Local ablation	35 (17.8)
TACE	66 (33.5)
Local ablation + TACE	34 (17.3)
Best support	54 (27.4)

*IQR*, interquartile ranges; *TACE*, transarterial chemoembolization

## Discussion

In the present study, we evaluated the short-term and long-term outcomes in a cohort of 239 consecutive patients with HCC and PH who underwent simultaneous splenectomy and curative treatment. The low perioperative mortality, low morbidity, remarkable improvement in liver function 1 year after splenectomy and a high long-term survival suggested that curative treatments for HCC and splenectomy for PH offer an alternative option for these patients.

Whether patients with Child B grade liver function should perform surgical treatment are still controversial. Almost half of the patients in our study are Child B grade liver function, and both of them underwent aggressive surgical treatments. The long-term outcome is different, but the perioperative outcome is acceptable, showing that surgical treatments are feasible. To clarify which patients could benefit from this surgical procedure, as well as decrease the bias and confounding, we took into account the factors that may affect long-term prognosis as much as possible. Multivariable analysis revealed that tumor size, tumor number, and Child score were independent risk factors for long-term survival. In spite of the fact that all patients had limited tumor burden, the prognosis of patients with larger or multiple tumors was relatively poor. This finding indicates that patients with great tumor burden benefit very little from this surgical procedure. Even though the Child score has several limitations in evaluating the preoperative liver function reserve,^22^ it remains the most frequently used tool to assess liver function reserve. We evaluated the OS in patients with different Child scores and found that a high Child score was a strong independent risk factor for poor prognosis. Although patients enrolled in this study had limited tumor burden, some patients had a poor prognosis, showing that the preoperative liver function was an important prognostic factor. Using the Child score system, we divided all patients into 5 subgroups and found that patients with Child score 8 or 9 had an inferior long-term survival, indicating that patients with limited tumor burden and relatively good liver function reserve could benefit from splenectomy combined with curative treatments, and liver transplantation should be the optimal selection for patients with limited tumor burden but poor liver function reserve.

High incidence of liver-related surgical complications, such as postoperative liver failure, delayed surgical site bleeding, and overloaded hydrothorax, is observed in cirrhotic patients.^25^ Postoperative liver failure is a troublesome and potentially life-threatening complication that occurs 1.2–32% of the time, especially in the cirrhotic background.^26^ In the current study, even though the overall morbidity as high as 75.3%, the PHLF rate was 3.3%, which was lower than most previous reports. The total mortality in our study was 2.1%: 5 patients died after surgery within 30 days. Our relatively low incidence of postoperative liver failure and perioperative mortality were mainly due to strictly preoperative patient selection, solid intraoperative hemostasis technique, and flexible postoperative management. In addition, most patients received minor hepatectomy to decrease the risk of postoperative liver function decompensated. Given that liver tumor resection could remove much functional liver parenchyma, patients with severe cirrhosis or small tumor (the largest tumor diameter less than 3 cm) but located deep in the liver parenchyma should not undergo liver resection as the first therapeutic option; rather microwave ablation is the optimal selection.^10^ The laparoscopic technique can also minimize the interruption of the portosystemic collateral vessels because only several trocar incisions in the anterior abdominal wall are enough to operate. The rate of liver failure and the overload of ascites after this procedure in patients with severe cirrhosis are lower.^27^ However, only a small proportion of patients in our study received laparoscopic surgery, and the benefit of the laparoscopic technique in patients with cirrhosis and PH should be further evaluated in a large cohort. Poor liver function and coagulation dysfunction also made it difficult to control the intraoperative blood loss, resulting in more blood loss and blood transfusion. In our study, the proportion of patients with an estimated intraoperative blood loss and an intraoperative transfusion volume were also more significant than previous studies.^16–19^ The portion of postoperative transfusion is also very high: as high as 58.6% of all patients received a transfusion. This result was attributed to a high percentage of patients with Child grade B liver function. Patients who needed a postoperative transfusion in this study usually had hypoalbuminemia and prolonged prothrombin time, resulting in overloaded ascites and delayed surgical site bleeding. Transfusion is a necessary and useful measure to correct hypoalbuminemia, reduce the overload of ascites, and improve the coagulative function.

Most HCCs develop in the setting of liver cirrhosis and are accompanied by PH, splenomegaly and liver decompensation, resulting in an impaired liver function and terrible performance status, which are also potentially lethal factors. Curative treatments can remove the liver tumor, but the cirrhotic liver background is not so natural to reverse. The tendency of liver decompensation and risk of variceal bleeding will increase with the evolution of cirrhosis and PH. After liver resection, other measures to extend survival time should be taken to improve decompensated liver function and to decrease the variceal bleeding tendency. In one study, varices were presented in approximately 50% of patients with cirrhosis and up to 85% of patients with decompensated cirrhosis.^28^ The variceal bleeding rate for cirrhotic patients with gastroesophageal varices is approximately 10–15% per year, and the six-week mortality for this group of patients ranges between 15 and 25%.^29^ Splenectomy combined with or without pericardial devascularization has been a standard surgical method to prevent the variceal bleeding tendency for almost 40 years in patients with PH and splenomegaly and has proved useful in avoiding repeated bleeding and extending the estimated survival time. In our study, 104 patients received pericardial devascularization. Half of these patients had a history of variceal bleeding, and others had tortuous varices in endoscopy screening. Variceal bleeding during the perioperative period was present in 5 patients before they were discharged, and all 5 had a bleeding history, 3 of them accompanied by the presence of PVT. Both of them recovered after thrombolytic therapy.

PVT is a potentially lethal complication, and the incidence of PVT varies from 10 to 36% after splenectomy.^30–32^ In the present study, PVT was observed in 65 patients (27.2%). In our center, LMWH was injected subcutaneously on the third day after splenectomy to prevent PVT before it was detected. However, thrombolytic therapy and surgical site bleeding are two contradictory phenomena and are difficult to balance. Once the PVT was detected, thrombolytic treatment with a hefty dose of LMWH or oral warfarin was initiated immediately if no active bleeding was observed. Patients with PVT after splenectomy had an increasingly high tendency of variceal bleeding and liver failure due to the increased portal vein pressure and the decreased hepatic inflow. Given that anticoagulant was given after splenectomy, no PHLF secondary to PVT was observed, and only five patients had transient variceal bleeding from the presence of PVT, and all five patients recovered smoothly with conservative therapy.

A series of previous reports have demonstrated that patients could benefit from splenectomy, especially for those with a Child grade B liver function.^12,13^ Once the hypertrophic spleen was removed, portal vein inflow reduced 20–30%, thereby significantly decreasing the portal pressure and the decompensated liver function, and the coagulation dysfunction can be markedly improved, resulting in good performance status and long-term survival.^33,34^ In our study, the routine blood test, coagulation, and liver function were successfully tested in 209 of 239 patients 1 year after splenectomy. We found that serum albumin level and prothrombin activity significantly increased, and although not significant, serum total bilirubin decreased, which resulted in a marked improvement of liver function in most Child grade B patients. Only a small proportion of patients still had a poor liver function. The Child classification improved in 98 of 101 patients (97%) who had been classified as Child grade B preoperatively. In the subgroup of patients with a preoperative Child A classification, even though the Child score and classification in most patients did not change 1 year after splenectomy, serum albumin increased significantly. Interestingly, liver function in 9 patients (8.3%) with preoperative Child A classification deteriorated into Child B classification 1 year after splenectomy. Decompensated liver function and hypersplenism preclude most aggressive therapies, such as liver resection, local ablation and TACE for HCC due to pancytopenia, hypoproteinemia and terrible performance status. Our study suggests that splenectomy combined with hepatectomy or local ablation not only removed the tumor but also promoted the amelioration of liver function, which was a significant factor for the aggressive therapy of recurrent liver tumor.

This study had several limitations. First, all of these cases were from only one center, and the sample was small, especially the proportion of patients with a Child score of 8 or 9. No comparison group without splenectomy also limited the interpretation of the results. Even though univariate and multivariate analysis had identified that splenectomy was a protective factor, selection bias and confounding factors may still exist. Second, the improvement of liver function 1 year after splenectomy may have been due to other reasons, such as antivirus therapy, nutrition therapy, and application of liver protective drugs. Our data do not allow any interpretation regarding the inner mechanism of liver function change. Therefore, cellular and molecular research is necessary to strengthen the role of splenectomy in liver function improvement. In addition, the postoperative liver function was evaluated only 1 year after splenectomy, and the long-term liver function change was not investigated. Since most of the survival information was obtained from telephone follow-up, the long-term change in liver function after splenectomy and curative treatment was difficult to record. To reduce these biases and get more long-term information, a large, randomized, controlled, rigorous study is needed to emphasize the role of synchronous splenectomy and curative treatment for short-term and long-term outcomes in patients with HCC and PH.

## Conclusion

Even though splenectomy combined with hepatectomy or local ablation to treat patients with HCC and PH has been performed for more than 20 years, this surgical procedure is still controversial and not widely accepted. Our study showed that splenectomy combined with liver resection or local ablation in patients with HCC and PH was safe and could achieve long-term survival, especially for patients with a limited tumor burden and Child scores of 5, 6, and 7. Liver function in most patients with preoperatively decompensated status showed significant improvement at 1 year after splenectomy. This surgical procedure is a viable surgical option for patients with limited tumor burden and Child scores of 5, 6, and 7 who could not undergo liver transplantation due to the shortage of liver donors.

## Electronic Supplementary Material

ESM 1 (DOCX 20 kb)
