# Supplementary material for: Benefits of Splenectomy and Curative Treatments for Patients with Hepatocellular Carcinoma and Portal Hypertension: a Retrospective Study
Source: J Gastrointest Surg. 2018 Dec 3;23(11):2151–62. doi: 10.1007/s11605-018-3981-9 (PMC6831533; doi:10.1007/s11605-018-3981-9)
Supplement: Supplementary file 1 — (DOCX 20 kb) [file 11605_2018_3981_MOESM1_ESM.docx]

Supplementary Table. Comparison of clinical data between the Child-Pugh A and B group

| Variables | Child A group  (n=123) | Child B group  (n=116) | *P* value |
| --- | --- | --- | --- |
| Age, years | 49.4±10.1 | 50.9±11.2 | 0.268 |
| Age＞65 | 7 (5.7) | 14 (10.3) | 0.233 |
| Sex |  |  |  |
| Male | 93 (75.6) | 96 (82.8) | 0.204 |
| Female | 30 (22.6) | 20 (17.2) |  |
| BMI | 22.2±2.5 | 22.3±2.2 | 0.659 |
| Etiology |  |  |  |
| Hepatitis B virus infection | 121 (98.4) | 109 (94) | 0.074 |
| Others | 2 (1.6) | 7 (6) |  |
| ASA score, points |  |  | 0.235 |
| ≤2 | 117 (95.1) | 105 (90.5) |  |
| ＞2 | 6 (4.9) | 11 (9.5) |  |
| Preoperative varices bleeding | 18 (14.6) | 34 (29.3) | 0.007 |
| Preoperative transfusion | 16 (13) | 24 (20.7) | 0.01 |
| Preoperative endoscopy therapy | 0 | 9 (7.8) | ＜0.001 |
| Esophageal varices |  |  | ＜0.001 |
| Small | 72 (58.5) | 28 (24.1) |  |
| Median | 22 (17.9) | 24 (20.7) |  |
| Large | 29 (23.6) | 64 (55.2) |  |
| ICG15 retention rates, % | 8.1±5.8 | 28.5±10.1 | ＜0.001 |
| WBC count, *10^12^/L | 3.0±1.2 | 3.0±1.5 | 0.606 |
| Platelet count, 10^3^/mm^3^ | 63.2±30.6 | 48.8±27.9 | ＜0.001 |
| Platelet count <10^3^/mm^3^ |  |  | 0.020 |
| No | 11 (8.9) | 2 (1.7) |  |
| Yes | 112 (91.1) | 114 (98.3) |  |
| Albumin (g/L) | 38.41±4.24 | 33.52±4.84 | ＜0.001 |
| Total bilirubin, μmol/L | 16.6±6.9 | 24.4±14.0 | ＜0.001 |
| AST, IU/ml | 39.1±27.3 | 45.2±32.6 | 0.057 |
| ALT, IU/ml | 41.6±41.4 | 41.4±32.0 | 0.993 |
| PT, second | 14.5±1.80 | 16.5±1.78 | ＜0.001 |
| INR | 1.23±0.13 | 1.42±0.18 | ＜0.001 |
| Ascites, Yes | 14 (11.4) | 96 (82.8) | ＜0.001 |
| MELD score, points | 7.8±2.9 | 10.4±3.2 | ＜0.001 |
| HBV-DNA > 10^4^ copy/mL | 32 (26) | 26 (22.4) | 0.549 |
| AFP, ng/ml |  |  | 0.040 |
| ≤400 | 84 (68.3) | 93 (80.2) |  |
| ＞400 | 39 (31.7) | 23 (19.8) |  |
| Spleen thickness, cm | 5.12±0.8 | 5.48±0.93 | 0.004 |
| Portal vein diameter, cm | 1.32±0.18 | 1.36±0.18 | 0.077 |
| Cirrhosis |  |  | 0.112 |
| Absence | 13 (10.6) | 5 (4.3) |  |
| Moderate and severe | 110 (89.4) | 111 (95.7) |  |
| Size of the largest tumor, cm | 3.86±1.92 | 3.5±1.74 | 0.142 |
| Tumor size, cm |  |  | 0.195 |
| ≤3 | 59 (48) | 66 (56.9) |  |
| ＞3 | 64 (52) | 50 (43.1) |  |
| Tumor number |  |  |  |
| Solitary | 117 (95.1) | 105 (90.5) | 0.211 |
| Multiple | 6 (4.9) | 11 (9.5) |  |
| Tumor differentiation^†^ |  |  | 0.252 |
| Well or moderately | 80 (75.5) | 52 (66.7) |  |
| Poorly | 26 (24.5) | 26 (33.3) |  |

BMI, body mass index; ASA, American Anaesthesia Association; ICG R-15, indocyanine green retention rates at 15 minutes; WBC, white blood cell; AST, aspartate transaminase; ALT, alanine transaminase; PT, prothrombin time; INR, international normalized ratio; MELD, Model for End-State Liver Disease; HBV-DNA, hepatitis B virus deoxyribonucleic acid; AFP, α-fetoprotein.

^†^ Only patients underwent liver resection had clinical pathology test (n=184).
